# Epigenetics and seasonal timing in animals: a concise review

**DOI:** 10.1007/s00359-023-01673-3

**Published:** 2023-09-11

**Authors:** Bettina Fishman, Eran Tauber

**Affiliations:** Department of Evolutionary and Environmental Biology, Institute of Evolution, University of Haifa, Haifa, Israel

**Keywords:** Photoperiodism, Epigenetics, Seasonal timing, DNA methylation, Histone modifications, ncRNA

## Abstract

Seasonal adaptation in animals is a complex process that involves genetic, epigenetic, and environmental factors. The present review explores recent studies on epigenetic mechanisms implicated in seasonal adaptation in animals. The review is divided into three main sections, each focusing on a different epigenetic mechanism: DNA methylation, histone modifications, and non-coding RNA. Additionally, the review delves into the current understanding of how these epigenetic factors contribute to the regulation of circadian and seasonal cycles. Understanding these molecular mechanisms provides the first step in deciphering the complex interplay between genetics, epigenetics, and the environment in driving seasonal adaptation in animals. By exploring these mechanisms, a better understanding of how animals adapt to changing environmental conditions can be achieved.

## Introduction

Epigenetics plays a significant role in the adaptation of organisms to their environment (Ashe et al. 2021). Epigenetic modifications are changes in gene expression that do not alter the underlying DNA sequence but can be passed down from one generation to the next. These modifications are influenced by a variety of factors, including environmental cues such as diet, stress, exposure to toxins, and seasonal change.

Basic theoretical models of epigenetic inheritance in both asexual and sexual organisms suggested that non-genetic inheritance can serve as a beneficial form of adaptive transgenerational plasticity in a dynamic environment (Jablonka et al. 1995; Edelaar et al. 2023). Specifically, if environmental conditions are subject to predictable fluctuations, parents can gain an advantage by producing offspring whose phenotypes are optimized for the expected conditions.

The annual cycle of seasonal change, with its predictable patterns of temperature, light, and food availability, has played a significant role in driving the evolution of seasonal timing mechanisms in both animals and plants (Visser et al. 2010). These mechanisms allow organisms to anticipate and adapt to the changing conditions of their environment, thereby optimizing their chances for survival and reproduction. This seasonal timer controls a wide range of physiological and behavioral processes, from migration and hibernation to reproduction and molting. Over time, these mechanisms have evolved in response to selective pressures such as competition for resources, predation, and changing climates, leading to a remarkable diversity of strategies for coping with seasonal change (Visser et al. 2010).

Seasonal adaptation is a complex process that involves both genetic and epigenetic factors. Studies in various organisms have suggested that both genetic and epigenetic factors play important roles in seasonal adaptation. However, the relative contribution of genetic and epigenetic factors may vary depending on the specific organism and the environmental cues involved. While genetic factors provide the foundation for seasonal adaptation, epigenetic modifications can fine-tune gene expression in response to changing environmental conditions (Ashe et al. 2021).

**Fig. 1:**
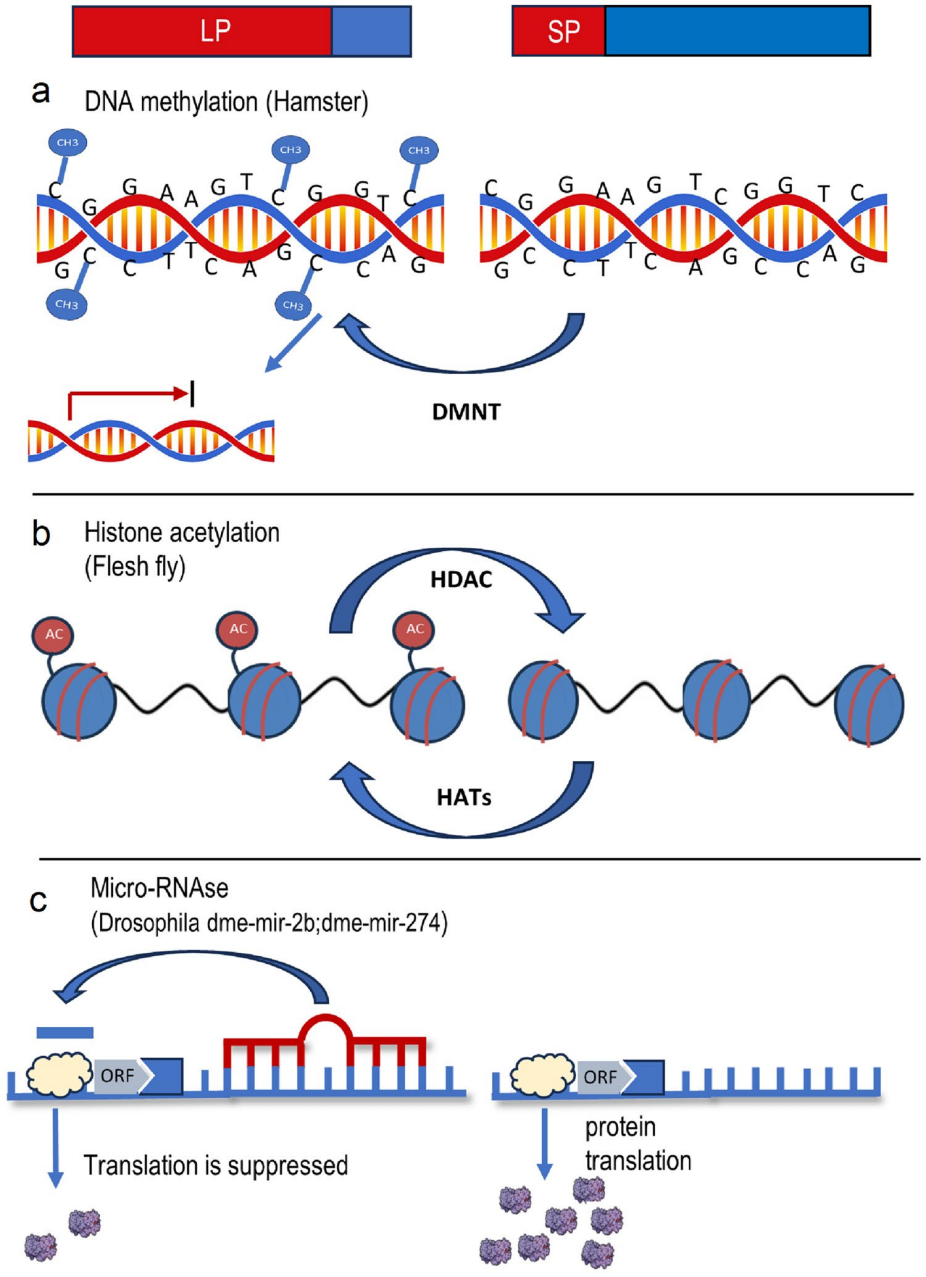
Epigenetic mechanisms associated with seasonal photoperiodic changes. Three representative examples are depicted of epigenetic responses to long (LP, left) and short (SP, right) photoperiods. **a** In the Siberian hamster, DNA methylation of CpG sites in the *dio3* promoter region under LP leads to attenuated transcription of the gene (Stevenson and Prendergast 2013). **b** In the flesh fly, reduced acetylation under SP is associated with global downregulation of transcription (due to compacted chromatin) and larval diapause (Reynolds et al. 2016). **c** In fruit-flies, higher expression of specific miRNA under LP leads to binding to their target transcripts and suppression of their translation (Pegoraro et al. 2022)

In this review paper, we will examine recent studies of epigenetic mechanisms implicated in seasonal adaptation in animals, which can be largely divided into three groups: DNA methylation, histone modifications, and non-coding RNA (Fig. 1). By exploring these mechanisms, we aim to provide insights into the complex interplay between genetics, epigenetics, and the environment in driving seasonal adaptation in animals.

## Epigenetic mechanisms

### DNA methylation

DNA methylation is a chemical modification that occurs on the DNA molecule, specifically on cytosine residues (Fig. 1). This modification involves the addition of a methyl group to the cytosine ring, resulting in the formation of 5-methylcytosine (5mC). DNA methylation is a crucial epigenetic mechanism that regulates gene expression and is involved in various cellular processes, such as development, differentiation, and genomic stability (Moore et al. 2012).

The process of DNA methylation is carried out by a family of enzymes called DNA methyltransferases (DNMTs). There are three types of DNMTs: DNMT1, DNMT2, and DNMT3. DNMT1 is responsible for maintaining the pattern of DNA methylation during DNA replication, while DNMT3a and DNMT3b are responsible for de novo methylation, meaning they add methyl groups to previously unmethylated cytosines In contrast, DNMT2, originally considered to be DNA methyltransferase is actually catalyzing the methylation of tRNA, (Jeltsch et al. 2017).

The process of DNA methylation occurs when the DNMT enzymes transfer a methyl group from the methyl donor molecule S-adenosyl methionine (SAM) to the carbon in position 5 of the cytosine ring. In animals, DNMTs predominantly recognize CpG dinucleotides (where cytosine and guanine are adjacent to each other in the DNA sequence) and add a methyl group to the cytosine residue.

The process of DNA methylation is reversible, and there are enzymes called demethylases that can remove the methyl group from 5mC to convert it back to cytosine. Ten-eleven translocation (TET) enzymes, so named due to their involvement in the translocation between chromosome 10 and chromosome 11, are responsible for the process of active DNA demethylation. This process involves the conversion of 5mC to 5-hydroxymethylcytosine (5hmC) and subsequent oxidation steps that eventually lead to the removal of the methyl group. In addition, demethylation may occur passively due to a lack of re-methylation after cell replication.

The parasitic wasp *Nasonia vitripennis*, commonly found in temperate zones exhibits a unique reproductive behaviour in response to short photoperiods (SP). Under autumnal SP, *N. vitripennis* females play a crucial role as mothers by laying eggs that will later undergo developmental arrest in the larval stage, resulting in diapause. The diapause is averted under long photoperiods (LP). This adaptation enables the species to survive the winter. The transgenerational transfer of the photoperiodic information from mothers to their offspring alluded to an epigenetic mechanism, which was shown to involve DNA methylation (Pegoraro et al. 2016). *Nasonia* has a complete mammalian-like kit of DNA methylation machinery (Werren et al. 2010) including all three DNA methyltransferases (Dnmt1-3), suggesting that methylation has a role to play in the life-history of this species, like in other hymenopteran insects (Lyko et al. 2010). Indeed, sequencing of bisulphite-treated DNA reveals that DNA methylation is prevalent and occurs at CpG sites as in mammals (Park et al. 2011). This is in sharp contrast to *Drosophila*, where only a single DNMT2 is present and methylation is rare, occurring at CpT or CpA sites (Raddatz et al. 2013).

DNA methylation analysis using reduced representation bisulphite sequencing (RRBS) revealed 31 genes whose methylation differed between SP and LP (Pegoraro et al. 2016). Given the limited CpG coverage by RRBS, it is estimated that the actual number of differentially methylated genes is 20-fold higher. Importantly, experiments knocking down *Dnmt1a* and *Dnmt3* by dsRNAi injections, or blocking DNA methylation pharmacologically, disrupted the photoperiodic response of the wasps. Specifically, following the RNAi knockdown, the diapause incidence increased under LP, and after the pharmacological treatment the diapause increased in LP and decreased in SP. Both these experiments indicated the causal role of DNA methylation.

DNA methylation was also implicated in the mammalian photoperiodic response. The thyroid hormone (T4) plays an important signalling role in the hypothalamus in transmitting photoperiodic information to the reproductive neuroendocrine system. Changes in photoperiod regulate the expression of the enzyme deiodinase type II (DIO2) that convert T4 to either the active T3, or the enzyme deiodinase type III (DIO3) that renders the receptor inactive, creating a seasonal gating mechanism for thyroid hormone receptor signalling (Dardente et al. 2014).

The hormone melatonin, produced by the pineal gland, also plays a crucial role in the regulation of the circadian system and photoperiodic responses. Melatonin is a neurohormone that is synthesized and released in a circadian rhythm, primarily during the dark phase of the day. Its production is under the direct control of the SCN. Pineal melatonin is not only essential for the control of circadian rhythms but also plays a vital role in mediating the effects of photoperiod on the reproductive neuroendocrine system. It is necessary for the induction of *dio3* mRNA expression by photoperiod (Ono et al. 2008), which acts as a molecular switch for the seasonal control of reproduction. Melatonin levels, in conjunction with the photoperiodic information received by the SCN, modulate the expression of *dio3*, influencing the activity of the thyroid hormone receptor signalling pathway.

A study on Siberian hamsters (*Phodopus sungorus*) with seasonal breeding patterns found that DNA methylation of the *dio3* promoter was critical in regulating *dio3* expression (Stevenson and Prendergast 2013). The study found that under LP, *dnmt3b* was expressed at higher levels, resulting in a reduction in *dio3* mRNA levels. Specifically, methylation was detected in 85% of 17 CpG sites in the *dio3* promoter region of hamsters under LP, compared to 42% under SP. The reduced *dio3* promoter methylation in SP was associated with up-regulation of *dio3* expression, which in turn resulted in gonadal regression (Stevenson and Prendergast 2013).

As previous studies have shown evidence for circadian changes in DNA methylation (Azzi et al. 2014), a follow-study was carried out to investigate whether the circadian patterns of hypothalamic *dnmt*s transcripts are influenced by the seasonal time (Stevenson 2017). The results showed that expression of *dnmt3a* (and to a lesser extent *dnmt1*, *dnmt3b*) was significantly greater in LP compared to SP at multiple points along the circadian period.

A study by Lindner et al. (2021) demonstrated the involvement of DNA methylation in seasonal timing in the great tit (*Parus major*). Several loci were identified where the level of promoter methylation was associated with the reproductive timing of the females. The most interesting gene was NR5A1, which codes for a transcription factor that regulates the expression of many key genes within the reproductive axis. Three CpG sites in the promoter region of NR5A1 showed a substantial increase in DNA methylation with the reproductive timing of the females.

The Melatonin receptor 1A (MTNR1A) is a pivotal gene involved in regulating the estrus cycle and seasonal reproduction in sheep. Recent investigations conducted in ewes by He et al. (2023) revealed that changes in photoperiod trigger DNA methylation at a specific cytosine site within the core promoter region of the MTNR1A gene. Moreover, an elevation in DNA methylation levels was observed following the long photoperiod (LP), demonstrating a significant negative correlation with the expression of MTNR1A.

In contrast to the circadian pacemaker, which is relatively resistant to temperature changes, the photoperiodic timer is highly sensitive to thermal modulation (Tyukmaeva et al. 2020). DNA methylation may play a crucial role in the integration of the photoperiodic timer with temperature changes. A recent study on redheaded buntings *Emberiza bruniceps* (Trivedi et al. 2019) proposed that DNA methylation mediates this integration. In long-day breeder songbirds, including the male redheaded buntings, exposure to LP triggers testicular enlargement, which is amplified at higher temperatures. The hypothalamus-pituitary–gonadal (HPG) pathways are involved in the regulation of gonadal growth and development, which is photoinduced under LP.

### Histone modifications

Histones are proteins that help package DNA into a compact structure in the nucleus. Modifications to histones, such as acetylation and methylation (Fig. 1), can affect how tightly the DNA is packaged and therefore its accessibility to the transcriptional machinery (Millán-Zambrano et al. 2022). Acetylation is the addition of an acetyl group to the lysine residues in histones. This modification typically leads to a more open chromatin structure and increased gene expression. Acetylation can neutralize the positive charge on histones, reducing their ability to interact with negatively charged DNA and making the DNA more accessible to transcription factors (Rothbart and Strahl 2014).

Methylation is the addition of a methyl group to lysine or arginine residues in histones. This modification can either activate or repress gene expression, depending on the specific residue that is methylated and the number of methyl groups that are added. For example, methylation of lysine 4 on histone H3 (H3K4me) is generally associated with active gene expression, while methylation of lysine 9 on histone H3 (H3K9me) is associated with gene silencing (Vinci 2012).

A study in the flesh fly *Sarcophaga bullata* suggested that histone modification might be a type of epigenetic process that contributes to the regulation of pupal diapause (Reynolds et al. 2016). The total acetylation of histone H3 was significantly reduced in early diapause compared to same-stage pupae that did not undergo diapause. Furthermore, a reduction in transcription of *gcn5*, a gene encoding histone acetyltransferases (HAT), which is responsible for the decrease in histone H3 acetylation. Additionally, first-instar larvae in diapause show changes in genes and enzymes associated with histone acetylation/deacetylation, including a 1.8-fold increase in transcription of *tip60* and *reptin*, genes encoding parts of the Tip60 HAT complex, and a substantial downregulation of histone deacetylases (HDAC) *hdac3* and *sirt1* in diapausing pupae. Overall, these results suggest that changes in histone H3 acetylation, as well as shifts in HAT and HDAC abundance and activity, are associated with diapause in *S. bullata* and have a significant impact on the programming, maintenance, and termination of diapause.

A similar global repression of transcription, driven by histone deacetylation, was also found in hibernating mammals. In thirteen-lined ground squirrels (*Ictidomys tridecemlineatus*), torpor was accompanied by attenuation of transcription, which, in turn, is associated with histone H3 acetylation (Biggar and Storey 2014). Studies in the Siberian hamster indicated that expression of *hdac1*, *hdac2*, and *hdac3* was regulated differently by the photoperiod and in a tissue-dependent manner (Lynch et al. 2017). For example, *hdac3* expression in the uterus increased in LP, while hdac2 was reduced. In the testes, *hdac1* is downregulated under SP, while *hdac3* is upregulated.

Histone methylation also serves as a regulator of photoperiodic diapause. In the cotton bollworm *Helicoverpa armigera*, under SP, there is a low level of tri-methylation of lysine 27 on histone H3 protein (H3K27me3). This represses the prothoracicotropic hormone (PTTH) gene expression, causing pupal diapause and lifespan extension (Lu et al. 2013). Similarly, differences in levels of H3K27me2 and H3K4me3 were associated with embryonic diapause in annual killifish (Toni and Padilla 2016).

In *Drosophila melanogaster*, active chromatin marks H3K4me3 and H3K36me1 were found to be reduced in diapausing ovaries, promoting diapause plasticity (Evans et al. 2022). The study also revealed that chromatin determinants of diapause plasticity may be genotype-dependent, indicating genetic variation for epigenetic regulation of reproductive diapause.

### Non-coding RNA

Non-coding RNA molecules are RNA molecules that do not encode for proteins, but instead have regulatory roles in gene expression. A significant class of non-coding RNAs, is microRNA (miRNA), which regulates gene expression at the post-transcriptional level (Bartel 2018). miRNA are small molecules, typically consisting of 18 to 25 nucleotides, which are involved in the RNA interference machinery, binding to the untranslated regions (UTRs) of mRNA molecules to suppress or modulate protein translation or induce mRNA decay (Fig. 1). A single miRNA has the potential to target numerous mRNAs, suggesting a global impact on the transcriptomes and proteomes of eukaryotes. Additionally, miRNAs can function as epigenetic modulators by targeting key enzymes that catalyse epigenetic reactions, such as DNMTs and HDACs (Yao et al. 2019). Furthermore, miRNA expression itself is subject to regulation by epigenetic machinery, including DNA methylation, RNA modification, and histone modification (Yao et al. 2019).

A recent study in *Drosophila* demonstrated the important role of miRNA in photoperiodic timing (Pegoraro et al. 2022). The comparison of miRNA expression in the head under SP and LP revealed seven miRNAs that were differentially expressed. Furthermore, overexpression of three of these miRNAs (*dme*-mir-2b, *dme*-*mir-184*, and *dme-mir-274*) in clock neurons in the brain, using the binary UAS/Gal4 system, led to a similar diapause response in SP and LP, suggesting that these miRNAs have functional roles in photoperiodic timing. Additionally, by utilizing both computational prediction and immunoprecipitation of RNA samples with the Argonaute-1 antibody from flies in LP and SP, the targets of photoperiodic miRNAs have been identified.

In a recent study conducted in sheep, the regulation of chromogranin A (CHGA) by non-coding RNA was identified (Di et al. 2023). CHGA was found to be upregulated in the pituitary pars tuberalis (PT), which plays a crucial role in encoding the photoperiodic response. Specifically, miR-25 was observed to bind to the 3' UTR region, resulting in the inhibition of CHGA expression. Additionally, miR-25 also targets a long non-coding RNA (lnc107153).

During the LP, there was a high expression of miR-25 in sheep, leading to the suppression of CHGA protein expression. However, in SP, the expression of Lnc107153 was found to be elevated, facilitating its binding to miR-25. This interaction weakened the inhibitory effect of miR-25 on CHGA expression. Consequently, there was a significant increase in the expression level of CHGA protein during SP.

Another class of non-coding RNAs is lncRNAs. These molecules are longer than 200 nucleotides and are not translated into proteins. While they do not encode proteins, they are involved in various cellular processes, including gene expression regulation (Reviewed by Statello et al. 2020). lncRNAs exhibit various interactions with DNA, other noncoding RNAs, and proteins, contributing to a wide array of modifications at transcriptional, post-transcriptional, and post-translational levels. They perform diverse roles such as serving as guides, decoys, and scaffolds, and participating in splicing, messenger RNA (mRNA) decay, and subcellular localization. Additionally, lncRNAs can impact chromatin architecture by engaging with chromatin-modulating proteins, thereby controlling transcriptional activity. This control can involve either promoting or preventing the recruitment and association of these proteins with chromatin. Through this mechanism, lncRNAs can either activate or repress transcription by recruiting regulatory factors to specific loci and modulating their functions (Reviewed by Sun et al. 2018).

A transcriptomic study (Nakayama et al. 2019) comparing SP and LP in the Japanese medaka fish (*Oryzias latipes*) revealed a photoperiodic lncRNA that was named a long-day-induced anti-sense intronic RNA (LDAIR). In transgenic fish where LDAIR has been knocked out, the expression of several neighboring genes has been altered. This neighborhood effect (common for lncRNA) has included the corticotropin-releasing hormone receptor 2 (CRHR2), known to be involved in the stress response. In line with this, under LP conditions (when LDAIR is expressed), wild-type medaka fish exhibited higher 'protective' diving behavior and increased light avoidance compared to knockout fish (Nakayama et al. 2019).

A recent study in sheep (Wang et al. 2022) identified 664 lncRNAs in the thyroid gland whose expression differed between short photoperiod (SP) and long photoperiod (LP). Co-expression network analysis suggested that some of these lncRNAs target genes such as CCNB3 and DMXL2, both of which are involved in reproduction.

## Epigenetic regulation of the circadian clock

Long before the molecular details of the circadian clock were elucidated, it was hypothesized that the 24-h pacemaker is an essential component of photoperiodic timing (Bünning 1967). Various models and experimental protocols such as the Nanda-Hamner or Bünsow resonance experiments, have been suggested for testing the link between the circadian clock and seasonal timing (reviewed by Tauber and Kyriacou 2001).

The role of the circadian clock in detecting seasonal environmental changes has also been implied by neural analysis of the suprachiasmatic nucleus (SCN), the mammalian principal pacemaker in the brain (Evans et al. 2013). Neurons in the two sub-regions of the SCN, the 'shell' and the 'core', differ in the phase of local circadian gene expression. This phase divergence varies as a function of the length of the photoperiod and may serve as a seasonal timer. A similar proposition was made in *Drosophila*, where two clock neuron clusters that regulate morning and evening diurnal activity, respectively, encode the seasonal change in photoperiod (Stoleru et al. 2007).

The molecular circuitry underlying the circadian clock is based on a set of transcriptional-translational feedback loops (TTFLs) that involve several key genes and their protein products (Reviewed by Takahashi 2016). The core TTFL of the circadian clock in animals involves a set of transcriptional activators and repressors that interact with each other in a negative feedback loop. In *Drosophila*, the activators include the transcription factors CLOCK (CLK) and CYCLE (CYC; the vertebrate ortholog is called BMAL1), which form a heterodimer that binds to specific DNA sequences known as E-boxes in the promoters of target genes (Allada et al. 1998; Rutila et al. 1998). The repressors include the proteins PERIOD (PER) and TIMELESS (TIM), which form a complex that inhibits the activity of the CLOCK-CYC complex (Sehgal et al. 1994).

The circadian TTFL in *Drosophila* and mammals is similar, with a key difference in CRY, which is a core clock protein in mammals rather than a photoreceptor in *Drosophila*. CRY takes on the role of TIM, serving as the partner of PER in mammals (the role of the mammalian TIM in the clock is disputed).

The CLOCK-BMAL1 complex activates the transcription of several genes, including the ones encoding the PER and CRY proteins. Subsequently, the PER and CRY proteins are synthesized and form a complex that inhibits the activity of the CLOCK-BMAL1 complex, thereby completing the negative feedback loop. This feedback loop results in oscillations in the levels of the PER and CRY proteins, which drive the oscillations in the activity of the CLOCK-BMAL1 complex and the expression of circadian rhythms (Reviewed by Takahashi 2016).

The TTFL oscillation is supported by accessory loops in which CLOCK and BMAL1 drive E-box-mediated circadian expression of the nuclear receptors RORα, REV-ERBα, and REV-ERBβ. These nuclear receptors, in turn, act via REV response element (RRE) sequences to activate and suppress BMAL1 transcription, respectively.

The identification of clock genes allowed direct testing of the link between the circadian and the seasonal clocks. As expected, knocking down of clock genes disrupted the photoperiodic response in insects such as the bean bug *Riptortus pedestris* (Ikeno et al. 2010), silkworm *Bombyx mori* (Tobita and Kiuchi 2022), band-legged ground cricket *Dianemobius nigrofasciatus* (Goto and Nagata 2022) and *Nasonia vitripennis* (Dalla Benetta et al. 2019). Importantly, natural genetic variation in circadian clock genes has been associated with variation in the seasonal response, suggesting that the circadian system is one of the targets of evolution for local adaptation of seasonal timing (Tauber et al. 2007; Mathias et al. 2007; Paolucci et al. 2016).

Given the link between the circadian and the photoperiodic circuits, any epigenetic changes that influence the circadian periodicity will potentially impact the photoperiodic response. Various epigenetic mechanisms have been shown to affect circadian gene expression, including DNA methylation (Azzi et al. 2014) and histone acetylation (Etchegaray et al. 2002), and methylation (Katada and Sassone-Corsi 2010). Non-coding RNA has also been shown to regulate circadian expression (Reviewed by Pegoraro and Tauber 2008).

An example of a circadian clock gene that undergoes seasonal epigenetic regulation is *Clk*. An evolutionarily conserved domain in the protein consists of a long polyglutamine stretch (poly-Q) whose length is polymorphic in wild populations, in a broad range of organisms. The frequency of the poly-Q alleles has been found to vary depending on the latitude. In the Chinook salmon, the poly-Q variation covaries with a cline in the timing of spring migration in various western North American rivers (O’Malley and Banks 2008). A comparable clinal pattern of variation in *Clk* allele frequencies has been reported among geographic populations of several migratory bird species, where a covariation between the poly-Q polymorphism and timing of breeding and migration has been found (Bazzi et al. 2015; Saino et al. 2017; Justen et al. 2022).

Studies of DNA methylation of a CpG site flanking the poly-Q in barn swallow females found a strong correlation with the timing of breeding or migration (Saino et al. 2017, 2019). There was also a strong parent–offspring correlation in methylation at this locus that supported a process of intergenerational epigenetic inheritance and was argued to facilitate a rapid seasonal response to climate change (Saino et al. 2019). However, it is not yet known to what extent DNA methylation impacts the function of the circadian clock in a way that would alter the critical daylength. In addition, it is possible that the seasonal effects of *Clk* polymorphism and DNA methylation reflect a pleiotropic effect of this gene, as has been suggested for the effect of other clock genes on insect diapause (Bradshaw and Holzapfel 2010).

## Discussion

The adaptation of animals to seasonal changes is a complex process that involves the intricate interplay between genetics, epigenetics, and the environment. Genetic factors play a fundamental role in shaping an organism's ability to adjust to seasonal variations (Kyriacou et al. 2008). Variations in key genes involved in the circadian clock, photoperiodic response, and hormone signaling pathways can influence an individual's sensitivity and responsiveness to changing environmental cues. However, the genetic blueprint alone is not sufficient to fully explain the diverse and fine-tuned seasonal adaptations observed in animals. Epigenetic modifications, as described earlier (see Table 1 for a summary), provide an additional layer of regulation that facilitates gene expression patterns in response to environmental cues. These modifications can act as molecular switches, enabling phenotypic plasticity and facilitating rapid adaptation to changing seasonal demands, which cannot be solely accounted for by the selection of genetic variants (Carneiro and Lyko 2020). While the process of selection may take a considerable number of generations and require a large effective population size, epigenetic adaptation can be effective within a single generation. In animal species expanding their range to higher latitudes, populations at the edge of the species distribution face extreme seasonal fluctuations. The genetic variation in these edge populations is low (Eckert et al. 2008), thereby limiting their adaptive potential through new gene variants. In such cases, epigenetics is expected to be the primary mode of adaptation. A similar dynamic has been proposed for invasive species (Carneiro and Lyko 2020).

**Table 1:** Epigenetic mechanisms associated with seasonal timing

Epigenetic mechanism	Taxonomic group	Species	Target (gene, or genomic	Seasonal response	References
DNA methylation	Insects	*Jewel wasp* (*Nasonia vitripennis*)	CpG sites (genomic)	Larval diapause induced by SP (maternal)	Pegoraro et al. (2016)
	Mammals	Hamster (*Phodopus sungorus*)	CpG in the dio3 promoter	under LP *dio3* mRNA levels were reduction	Stevenson and Prendergast (2013)
	Mammals	Sunite sheep (*Ovis aries*)	CpG in MTNR1A promoter	Activation of the reproductive system in SP	He et al. (2023)
	Aves	Red-headed bunting (*Emberiza bruniceps*)		LP triggers testicular enlargement	Trivedi et al. (2019)
	Aves	Barn Swallow (*Hirundo rustica*)	CpG site flanking the poly-Q in *Clk*	Timing of breeding or migration	Saino et al. (2017, 2019)
	Aves	Great tit (*Parus major*)	CpG in NR5A1	Timing of reproductive season	Lindner et al. (2021)
Histone modifications	Insects	Flesh fly (*Sarcophaga bullata*)	Histone H3 HAT and HDAC	regulation of pupal diapause	Reynolds et al. (2016)
	Insects	Fruit fly (*Drosophila melanogaster*)	H3K4me3 and H3K36me1	Reproductive diapause in SP	Evans et al. (2022)
	Insects	Cotton bollworm (*Helicoverpa armigera*)	methyl modification (H3K27me3)	under SP, pupal diapause	Lu et al. (2013)
	Fish	Annual killifish (*Austrofundulus limnaeus*)	H3K27me2 and H3K4me3	embryonic diapause	Toni and Padilla (2016)
	Mammals	Ground squirrel (*Ictidomys tridecemlineatus*)	Histone H3 acetylation	torpor	Biggar and Storey (2014)
Non-coding RNA	Insects	Fruit fly (*Drosophila melanogaster*)	*dme-mir-2b*, *dme-mir-184*, and *dme-mir-274*	Reproductive diapause in SP	Pegoraro et al. (2022)
	Mammals	Sunite sheep (*Ovis aries*)	*miR-25 binding to lnc107153 and CHGA*	Activation of the reproductive system in SP	Di et al. (2023)
	Mammals	Sunite sheep (*Ovis aries*)	*lncRNA (various)*	seasonal reproduction	Wang et al. (2022)
	Fish	Medaka fish (*Oryzias latipes*)	*lncRNA LDAIR*	self-protective behaviours in LP	Nakayama et al. (2019)

The extent to which epigenetic mechanisms directly regulate photoperiodic timing remains unclear. To determine whether epigenetic modifications have a causal role in seasonal timing, a transgenic approach could be employed to test the effects of different epialleles in congenic animals. Consider, for example, the three CpG sites located in the proximal promoter region of *dio3* in the hamster, which have been found to exhibit differential methylation associated with the photoperiodic response (Stevenson and Prendergast 2013). To investigate the causal role of this methylation in seasonal timing, a potential approach could involve generating transgenic hamsters in which the *dio3* allele is replaced by a mutated allele lacking these specific CpG sites.

By employing such a transgenic model, it would be possible to directly test the effects of these CpG sites and determine whether their presence or absence influences the photoperiodic response. This approach holds the potential to elucidate the functional significance of the observed methylation patterns and establish a causal relationship between the epigenetic modifications and the regulation of seasonal timing.

One of the primary challenges in elucidating the role of epigenetics in seasonal timing is the lack of understanding of the underlying molecular circuitry of the photoperiodic clock. Unlike the circadian clock, which is known to be governed by a universal core pacemaker circuit, no such canonical photoperiodic circuit may exist. As such, deciphering the mechanism of the photoperiodic clock may be challenging.

Even if such a canonical circuit exists, it is conceivable that the majority of epigenetic modifications governing photoperiodic timing adjustments are situated outside of the central photoperiodic timer. This parallels the functioning of the circadian clock, where genetic variations influencing chronotype are predominantly found in genes outside of the core pacemaker circuit, as indicated by a recent meta-analysis GWAS study (Jones 2019). Notably, out of the 351 loci associated with chronotype variation, only seven were located in circadian clock genes. Consequently, it is reasonable to anticipate that most of the epigenetic adaptations will be species-specific and unique to each seasonal response, thereby potentially hindering the identification of universal principles across different organisms.

While mapping seasonal epigenetic changes is relatively straightforward, understanding the mechanism that induces these changes is more challenging. In the case of the flesh fly *S. bullata*, where diapause is associated with a global downregulation of transcription, the response can be carried out merely by HAT downregulation. However, in other cases, the seasonal response is often associated with both up- and down-regulation, necessitating complex epigenetic regulation. In *Nasonia*, for example, the photoperiodic response is associated with both increases and decreases in DNA methylation at different loci (Pegoraro et al. 2016).

The mechanisms that determine why certain genes are methylated while others are not remain unclear. One possibility is that DNA methylation might be influenced by chromatin structure and the binding of transcription factors and other regulatory proteins. For example, some proteins can recruit DNMTs to specific regions of the genome through protein–protein interactions, while others can prevent or enhance DNA methylation by altering chromatin structure or modifying the activity of DNMTs (Cedar and Bergman 2009).

The deposition of DNA methylation and specific histone modifications seem to have a reciprocal influence on each other (Jin et al. 2011). Histone methylation can direct DNA methylation patterns, while DNA methylation can act as a template for the formation of certain histone modifications following DNA replication. Therefore, these epigenetic modifications appear to have a bidirectional relationship that can impact gene expression regulation.

In conclusion, epigenetic modifications play an important role in seasonal timing, with numerous different mechanisms present in different organisms and even within individuals. While the underlying molecular circuitry of the photoperiodic clock remains elusive, studying the epigenetic regulation of seasonal timing may provide new insights into this complex biological process.
